# Dehydratase mediated 1-propanol production in metabolically engineered *Escherichia coli*

**DOI:** 10.1186/1475-2859-10-97

**Published:** 2011-11-10

**Authors:** Rachit Jain, Yajun Yan

**Affiliations:** 1 Biochemical Engineering Program, Faculty of Engineering, University of Georgia, Athens, GA 30602 USA

## Abstract

**Background:**

With the increasing consumption of fossil fuels, the question of meeting the global energy demand is of great importance in the near future. As an effective solution, production of higher alcohols from renewable sources by microorganisms has been proposed to address both energy crisis and environmental concerns. Higher alcohols contain more than two carbon atoms and have better physiochemical properties than ethanol as fuel substitutes.

**Results:**

We designed a novel 1-propanol metabolic pathway by expanding the well-known 1,2-propanediol pathway with two more enzymatic steps catalyzed by a 1,2-propanediol dehydratase and an alcohol dehydrogenase. In order to engineer the pathway into *E. coli*, we evaluated the activities of eight different methylglyoxal synthases which play crucial roles in shunting carbon flux from glycolysis towards 1-propanol biosynthesis, as well as two secondary alcohol dehydrogenases of different origins that reduce both methylglyoxal and hydroxyacetone. It is evident from our results that the most active enzymes are the methylglyoxal synthase from *Bacillus subtilis *and the secondary alcohol dehydrogenase from *Klebsiella pneumoniae*, encoded by *mgsA *and *budC *respectively. With the expression of these two genes and the *E. coli ydjG *encoding methylglyoxal reductase, we achieved the production of 1,2-propanediol at 0.8 g/L in shake flask experiments. We then characterized the catalytic efficiency of three different diol dehydratases on 1,2-propanediol and identified the optimal one as the 1,2-propanediol dehydratase from *Klebsiella oxytoca*, encoded by the operon *ppdABC*. Co-expressing this enzyme with the above 1,2-propanediol pathway in wild type *E. coli *resulted in the production of 1-propanol at a titer of 0.25 g/L.

**Conclusions:**

We have successfully established a new pathway for 1-propanol production by shunting the carbon flux from glycolysis. To our knowledge, it is the first time that this pathway has been utilized to produce 1-propanol in *E. coli*. The work presented here forms a basis for further improvement in production. We speculate that dragging more carbon flux towards methylglyoxal by manipulating glycolytic pathway and eliminating competing pathways such as lactate generation can further enhance the production of 1-propanol.

## Background

The excessive utilization of petroleum plays a major role in the release of the green house gas-carbon dioxide contributing to global warming. Renewable energy sources provide a wide platform of resources to address the problem of increasing energy demand. The manufacture of biofuels such as higher chain alcohols from renewable sources provides an alternative energy source which possesses the advantage of having desirable fuel properties and uncomplicated transportability [1-4]. The synthesis of various higher chain alcohols has been achieved by constructing biosynthetic pathways in *E. coli *and other micro-organisms [1-7]. Here, we describe the design of a new pathway for 1-propanol synthesis and its validation in *E. coli*.

In petrochemical industry, 1-propanol is produced from ethene by a reaction with carbon monoxide and hydrogen to give propionaldehyde, which is then hydrogenated [8]. 1-propanol is also produced as a by-product when potatoes or grains are fermented during the commercial manufacture of ethanol [8,9]. The general use of 1-propanol is in the manufacture of drugs and cosmetics such as lotions, soaps, and nail polishes. It also finds applications in the manufacture of flexographic printing ink and textiles [8,9].

Recently, the use of 1-propanol as a potential fuel substitute to petroleum has promoted the interest in its production via biological approaches. In 2008, Atsumi *et al*. and Shen *et al*. reported the production of 1-propanol from glucose by metabolic engineering of *E. coli*. Their work relied on the keto-acid pathway in *E. coli *with 2-ketobutyrate as a key intermediate [1,7]. The 2-ketobutyrate was converted to 1-propanol by the action of a keto acid decarboxylase and an alcohol dehydrogenase. Wild type *E. coli *carrying this pathway was able to produce around 0.15 g/L of 1-propanol. With the elimination of the genes *metA*, *tdh*, *ilvB*, *ilvl *and *adhE *encoding the enzymes *o*-succinyltransferase, threonine dehydrogenase, acetohydroxy acid synthase and alcohol dehydrogenase respectively, the production of 1-propanol achieved was 1 g/L. Atsumi *et al*. [2] reported higher levels of 1-propanol production in *E. coli *using *cimA *encoding a citramalate synthase from *Methanoccus jannaschii*. They established a direct route for the conversion of pyruvate to 2-ketobutyrate. With the utilization of citramalate pathway and incorporating an evolutionary strategy based on growth they were able to overcome feedback inhibition by isoleucine. Using wild type *cimA *they achieved 0.3 g/L of 1-propanol production. With the development of *cimA *variants, the production of 1-propanol was 9 times higher compared to the wild type *cimA*.

**Figure 1:**
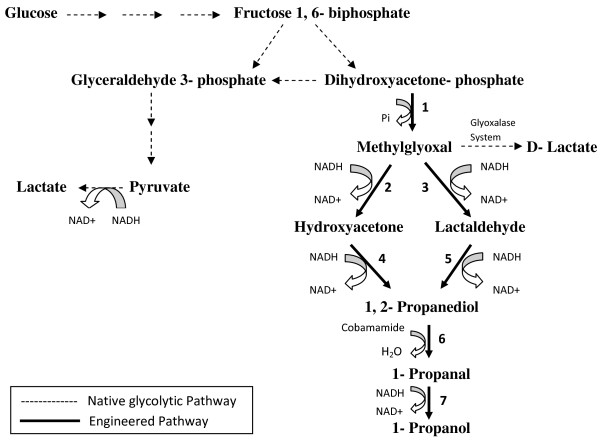
**Designed metabolic pathway for 1,2-propanediol and 1-propanol production**. Key enzymes 1: methylglyoxal synthase (*mgsA*); 2: methylglyoxal reductase (*ydjG*); 3, 4: secondary alcohol dehydrogenase (*gldA/budC*); 5: primary alcohol dehydrogenase (*fucO*); 6: diol dehydratase (*ppdABC/gldABC/dhaB12*); 7: primary alcohol dehydrogenase (*yqhD*).

We developed a new approach for the biosynthesis of 1-propanol by extending the well-known 1,2-propanediol pathway. As the pathway scheme shown in Figure 1, the intermediate of glycolysis dihydroxyacetone phosphate is converted to methylglyoxal by the action of the enzyme methylglyoxal synthase. The methylglyoxal generated is further reduced to either hydroxyacetone or lactaldehyde via two different routes. The formation of hydroxyacetone is catalyzed by the enzyme methylglyoxal reductase which is a primary alcohol dehydrogenase, while a secondary alcohol dehydrogenase such as glycerol dehydrogenase reduces methylglyoxal into lactaldehyde. Both hydroxyacetone and lactaldehyde can be further reduced to 1,2-propanediol by either a secondary alcohol dehydrogenase or a primary alcohol dehydrogenase. The dehydration of 1,2-propanediol into 1-propanal can be achieved by a diol dehydratase. The conversion of 1-propanal to 1-propanol is also catalyzed by a primary alcohol dehydrogenase.

The pathway that leads to the synthesis of 1,2-propanediol has been introduced into both *E. coli *and *Saccharomyces cerevisiae*. By over-expressing the *E. coli *genes *mgsA *and *gldA *and relying on the native expression of other enzymes, Altaras *et al*. achieved the production of 0.7 g/L of 1,2-propanediol in *E. coli *[10]. 1.08 g/L 1,2-propanediol production in *E. coli *was reported by Berrios-Rivera *et al*. by utlizing *Clostridium acetobutylicum mgsA *and *E. coli gldA *in a strain defecient in lactate production and using an initial glucose concentration of 101.68 mM [11]. Enhanced production of 1,2-propanediol in *E. coli *was also reported by Altaras *et al*. [12]. The study involved expression of more complete pathway by addition of *fucO *gene (1,2-propanediol oxidoreductase) responsible for the conversion of lactaldehyde to 1,2-propanediol and deletion of the competing pathway for lactate which involves the gene *ldhA*. Shake flask fermentation with the *ldhA- *strain carrying the pathway led to the production of 1,2-propanediol at a titer of 1.27 g/L, while fed-batch fermentation gave a result of 4.5 g/L of 1,2-propanediol. 1,2-propanediol production in *S. cerevisiae *was achieved by Joon-Young *et al*. [13]. Their strategy was based on the idea of channeling the carbon flux towards dihydroxyacetone phosphate with the deletion of triosphoshate isomerase in *S. cerevisiae *via triple homologous recombination. With the introduction of 1,2-propanediol pathways consisting of the *E. coli *genes *mgsA *and *gldA*, the engineered *S. cerevisiae *produced 1.11 g/L of 1,2-propanediol compared to 0.89 g/L produced from the strain lacking the gene *tpiI*.

In this study, we first constructed the 1,2-propanediol pathway in the wild type *E. coli *strain BW25113 by expressing the genes, *mgsA *from *B. subtilis*, *budC *from *K. pneumoniae*, and native *E. coli ydjG*, which resulted in the production of 1,2-propanediol at a titer of 0.8 g/L in shake flasks. We further achieved the conversion of 1,2-propanediol to 1-propanol via two successive enzymatic steps by expressing the operon *ppdABC *from *K. oxytoca *and using the native activity of *E. coli *alcohol dehydrogenases [14-16]. This established a new pathway for 1-propanol production by engineering the glycolytic pathway in *E. coli*.

## Results and Discussion

### Methylglyoxal Synthase Assay

1,2-Propanediol pathway branches from glycolysis and competes for the intermediate dihydroxyacetone phosphate, with the glycolytic pathway. The first enzyme of 1,2-propanediol pathway, methylglyoxal synthase catalyzing irreversible conversion of dihydroxyacetone-phosphate to methylglyoxal holds paramount importance in channeling carbon flux towards 1,2-propanediol biosynthesis [10,11]. Highly active methylglyoxal synthase is therefore desirable. We screened the activity of methylglyoxal synthase from eight different sources. We amplified the *mgsA *genes from the microorganisms: *C. acetobutylicum *(ATCC# 824), *B. subtilis *168, *C. difficile *R20291, *E. coli *MG1655, *T. thermophilus *HB27, *K. pneumoniae *MGH78578, *P. fluorescens *Pf-5, and *R. eutropha *H16 respectively. These genes were cloned and expressed in wild type *E. coli *BW25113 using eight plasmids pRJ1-pRJ8. Each gene was under the control of the IPTG-inducible pLlacO1 promoter. Using dihydroxyacetone-phosphate as the substrate, we successfully detected the functional expression of all *mgsA *genes *in vitro*, where the specific activities varied from 0.0052 U/mg to 0.1242 U/mg (Table 1). We identified most suitable methylglyoxal synthase as the *mgsA *from *B. subtilis *demonstrating the highest ratio of specific activity/*K_m _*(0.1186) and having a specific activity of 0.0561 U/mg. Without the over-expression of *mgsA *gene, we also detected the native expression of *E. coli mgsA*, which gave a specific activity of only 0.0008 U/mg, much lower than that of any over-expression.

**Table 1:** Methylglyoxal synthase assay results.

*mgsA *source	Specific Activity (U/mg)	*K_m _*(mM)	Specific Activity/*K_m _*(U/mg/mM)
*C. acetobutylicum*	0.054 ± 0.004	0.776 ± 0.005	0.069
***B. subtilis***	**0.056 ± 0.003**	**0.473 ± 0.07**	**0.118**
*C. difficile*	0.059 ± 0.0.003	1.439 ± 0.06	0.041
*E. coli*	0.124 ± 0.006	1.418 ± 0.12	0.087
*T. thermophilus*	0.016 ± 0.004	2.118 ± 0.07	0.007
*K. pneumoniae*	0.016 ± 0.009	2.820 ± 0.3	0.005
*P. fluorescens*	0.013 ± 0.008	1.560 ± 0.02	0.008
*R. eutropha*	0.005 ± 0.000	0.700 ± 0.03	0.007

Substrate dihydroxyacetone phosphate concentration was varied from 0.15 mM to 1.5 mM for all reactions. 1 unit (U) was defined as the amount (μmoles) of methylglyoxal formed per unit time (min).

### Methylglyoxal Reductase Assay

We examined the activity of *E. coli *methylglyoxal reductase encoded by the gene *ydjG *by using plasmid pRJ10. As a part of aldo-keto reductase family, the product of *ydjG *executes a catalytic activity of reduction on methylglyoxal using NADH to generate hydroxyacetone[17]. We determined both the specific activity and substrate affinity of *E. coli *methylglyoxal reductase on methylglyoxal. When the gene is over-expressed by pRJ10, the specific activity was determined to be 1.62 ± 0.012 U/mg. The enzyme also showed sufficient substrate specificity with a *K_m _*value of 3.31 ± 0.02 mM.

### Secondary Alcohol Dehydrogenase Assay

The synthesis of 1,2-propanediol from methylglyoxal occurs through two different pathways. For the pathway via lactaldehyde leading to 1, 2-propanediol formation we evaluated the activities of two NADH dependent secondary alcohol dehydrogenases: *E. coli *glycerol dehydrogenase (*gldA*) and *K. pneumoniae *diol dehydrogenase (*budC*) on methylglyoxal. We also tested the catalytic properties of these two secondary alcohol dehydrogenases on hydroxyacetone for the completion of the other pathway.

The genes *gldA *and *budC *were cloned and expressed in *E. coli *using the plasmid pRJ9 and pYY109. The specific activity and *K_m _*value of glycerol dehydrogenase and diol dehydrogenase were determined for the substrates methylglyoxal and hydroxyacetone. Both enzymes showed dehydrogenation activity leading to the conversion of methylglyoxal to lactaldehyde and hydroxyacetone to 1,-2-propanediol. Table 2 provides the results of this assay. The diol dehydrogenase and glycerol dehydrogenase reduced both methylglyoxal and hydroxyacetone. When methylglyoxal was used as the substrate, the diol dehydrogenase demonstrated a specific activity of 3.718 U/mg with a *K_m _*value of 0.78 mM; while the glycerol dehydrogenase showed both lower specific activity (2.456 U/mg) and substrate affinity (*K_m _*= 68.24 mM). Similar results were observed when hydroxyacetone was tested as a substrate, the diol dehydrogenase more efficiently reduced hydroxyacetone into 1,2-propanediol (specific activity = 4.97 U/mg; *K_m _*= 1.83 mM) compared with the glycerol dehydrogenase (specific activity = 0.912 U/mg; *K_m_*= 10.47 mM)

**Table 2:** Specific activity and *K_m _*determination of the secondary alcohol dehydrogenases.

Gene	Methylglyoxal		Hydroxyacetone	
	
	Specific Activity (U/mg)	*K_m_* (mM)	Specific Activity (U/mg)	*K_m_* (mM)
*gldA*	2.45 ± 0.00	68.24 ± 0.05	0.91 ± 0.00	10.47 ± 0.55
*budC*	3.71 ± 0.06	0.78 ± 0.03	4.97 ± 0.00	1.83 ± 0.63

The decrease in absorbance of NADH at 340 nm was recorded and used for calculations using the substrates methylglyoxal and hydroxyacetone. Substrate concentration was varied from 20 mM - 120 mM. 1 unit (U) was defined as the amount (μmoles) of product formed per unit time (min).

### Propanediol Dehydratase *in vivo *Assay

The diol dehydratases we tested included a propanediol dehydratase (PPD) originating in *K. oxytoca*, a glycerol dehydratase (GLD) from *K. pneumoniae*, and a glycerol dehydratase (GLD) from *C. butyricum*. The PPD of *K. oxytoca *and the GLD of *K. pneumoniae *are iso-functional enzymes which catalyze the coenzyme B12-depedent conversion of 1,2-propanediol or glycerol to the corresponding aldehyde [17-20]. These enzymes have been utilized to develop a biological process to produce 1,3-propanediol from glycerol [21]. Each of these enzymes consists of three subunits encoded by three structural genes (*ppdABC *or *gldABC*). Although the catalytic site is hosted by subunit A, the presence of subunits B and C are obligatory for enzyme activity [19]. In order to evaluate their catalytic efficiency towards 1,2-propanediol, all three subunits were co-expressed in *E. coli *to reconstitute the enzymes using the plasmids pYY93 and pYY134. The GLD from *C. butyricum *is a coenzyme B12-independent diol dehydratase comprised of two subunits encoded by *dhaB12*, which only demonstrates activity under strict anaerobic conditions [21]. To evaluate its catalytic efficiency, we constructed the plasmid pYY167 to co-express these two units.

The formation of 1-propanol from 1,2-propanediol involves two enzymatic steps. For the first step we evaluated the dehydration activity of three different diol dehydratases for the generation of 1-propanal. For the second step we relied on the native alcohol dehydrogenase activity of *E. coli *to convert the generated 1-propanal to 1-propanol. An experiment was designed and conducted as we described to perform the *in vivo *enzyme assay of propanediol dehydratase and also to evaluate the native activity of *E. coli *for the final step. Whole-cell bioconversion studies using wild type *E. coli *strain BW25113 carrying pYY93, pYY134, and pYY167 respectively were conducted in shake flasks by feeding 5 g/L (65.7 mM) 1,2-propanediol as the substrate. The samples were collected after 24 hours and analyzed by HPLC-RID.

**Figure 2:**
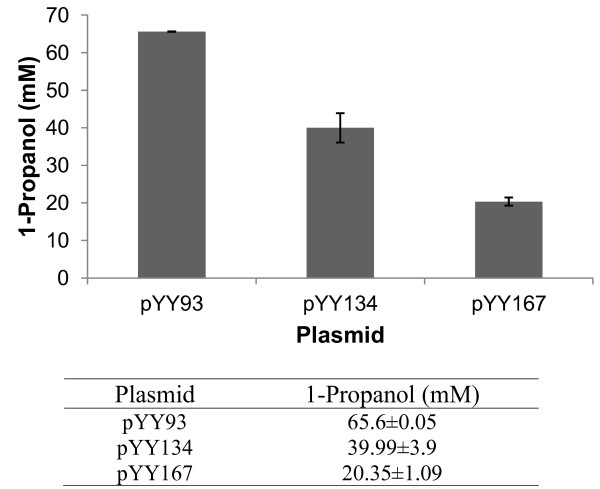
**The results of *in vivo *enzyme assay of diol dehydratase using 5 g/L (65.7 mM) 1,2-propanediol as the substrate**.

The results are presented in Figure 2. The catalytic efficiency of *K. oxytoca *PPD was the highest among all, producing 65.6 mM 1-propanol amounting to nearly 100% conversion. This result also indicated that the native expression of alcohol dehydrogenases in *E. coli *is sufficient to convert 1-propanal to 1-propanol completely. Over-expression of the alcohol dehydrogenases will not be necessary for 1-propanol production in *E. coli*. The *K. pneumoniae *GLD and *C. butyricum *GLD only demonstrated about 60.9% and 30.9% of the catalytic efficiency of *K. oxytoca *PPD, producing 39.99 mM and 20.35 mM 1-propanol, respectively.

### Production of 1,2-Propanediol and 1-Propanol in *E. coli*

In order to introduce the 1-propanol pathway into wild type *E. coli *strain BW25113, we constructed two plasmids. The first plasmid pRJ11 carries the genes encoding the most active enzymes for 1,2-propanediol biosynthesis. Specifically, *mgsA *from *B. subtilis*, *ydjG *from *E. coli*, and *budC *from *K. pneumoniae *were organized as a synthetic operon under the control of IPTG-inducible pLlacO1 promoter in a high-copy number plasmid. The second plasmid pYY93 contains only the structural genes *ppdABC *encoded by *K. oxytoca *PPD. For the enzymatic steps of lactaldehyde to 1,2-propanediol and 1-propanal to 1-propanol, we completely relied on the native expression of alcohol dehydrogenases in *E. coli*, as is indicated to be sufficient [10,11]. We also evaluated M9 media in comparison to low-phosphate media for the production of 1,2-propnaediol (Data not provided). The results of an initial experiment show significant increase in production using low-phosphate media. Using M9 media resulted in only about 0.1 g/L of 1,2-propanediol generation after 48 hours of anaerobic fermentation compared to about 0.8 g/L from low-phosphate media. Hence low-phosphate media was used for all fermentation studies. The culture conditions were the same as described in "Methods and Materials".

We first transformed the plasmid pRJ11 into wild type *E. coli *strain BW25113 to achieve 1,2-propanediol production. It has been reported that the enzyme methylglyoxal synthase is inhibited by phosphate ion [22,23]. The inhibition of methylglyoxal synthase would result in the carbon flux being diverted to glyceraldehyde-3-phosphate instead of methylglyoxal by the conversion action of triose phosphate isomerase. Therefore a low-phosphate media was employed to avoid this problem. The fermentation experiments were conducted in 20 ml cultures as described in the "Methods and Materials". The fermentation samples were collected after 24 hours and 48 hours and analyzed by HPLC-RID. As the results shown in Table 3, after 24 hours 0.66 g/L 1,2-propanediol was produced. The production reached 0.80 g/L after 48 hours. Lactate was detected as the dominant by-product and was accumulated at over 7 g/L. We also conducted the experiments aerobically. However, only a trace amount (< 0.01 g/L) of 1,2-propanediol was produced and the cell growth was much better than in anaerobic conditions, which indicates that glycolysis is very active in aerobic condition and drags almost all carbon flux towards pyruvate for cell growth or other cell activities.

**Table 3:** 1,2-Propanediol and 1-propanol production in low-phosphate media using *E.coli *strain *BW25113 *transformed with the appropriate plasmid(s)

Plasmid	1, 2-Propanediol (g/L)		1-Propanol (g/L)	
	24 h	48 h	24 h	48 h
pRJ11	0.66 ± 0.01	0.80 ± 0.01	-----	-----
pRJ11 and pYY93	0.44 ± 0.01	0.46 ± 0.02	0.11 ± 0.01	0.25 ± 0.07

With the successful establishment of the pathways for 1, 2-propanediol production using pRJ11, we hypothesized that the co-expression of pRJ11 with pYY93 would result in the production of 1-propanol. To test this, wild type *E. coli *BW25113 was transformed with both pRJ11 and pYY93 for 1-propanol production by electroporation. The fermentation condition was similar to that used for 1,2-propanediol production with the addition of 10 μM coenzyme B-12 to the culture along with IPTG (0.1 mM) after 6 hours. After 24 hours, the double transformed strain produced 0.11 g/L 1-propanol with 0.44 g/L 1, 2-propanediol remaining unconverted (Table 3). After 48 hours, 1-propanol was produced at 0.25 g/L with 0.46 g/L 1,2-propanediol remaining unconverted. The major by-product was again lactate at over 7 g/L.

## Conclusions

We have successfully established a new pathway for 1-propanol production by shunting the native glycolytic pathway in *E. coli*. The addition of the coenzyme B-12 dependent propanediol dehydratase from *K. oxytoca *resulted in the conversion of 1,2-propanediol to 1-propanal which was then dehydrogenated by *E. coli *native activity to 1-propanol.

From the assay of methylglyoxal synthase it was determined that the *mgsA *from *B. subtilis *was the most active. Since the accumulation of methylglyoxal in high quantities is toxic to the cell [24], it is important that the generated methylglyoxal is immediately converted to another metabolite by the downstream enzymes in the pathway. To address this problem we screened the activity of methylglyoxal reductase and two secondary alcohol dehydrogenases.

To evaluate 1, 2-propanediol formation, methylglyoxal synthase (*mgsA*) from *B. subtilis *and propanediol dehydratase (*budC*) from *K. pneumoniae *were expressed leading to the conversion of dihydroxyacetone phosphate to 1,2-propanediol via the formation of methylglyoxal and lactaldehyde. To strengthen our constructed pathway the introduction of *E. coli *methylglyoxal reductase (*ydjG*), a dual metabolic route for production of 1, 2-propanediol was established for the first time. This resulted in channeling the carbon flux from methylglyoxal to hydroxyacetone and 1,2-propanediol. Fermentation with *E. coli *BW25113 transformed with pRJ11 carrying the three above mentioned genes produced 0.8 g/L 1,2-propanediol after 48 hours of anaerobic fermentation.

We also evaluated the use of a medium copy number vector (pRJ12) for 1, 2-propanediol production (data not provided). This was done using the same genes used for the construction of high copy number plasmid pRJ11 but in the backbone of a medium copy number vector pCS27. However, the production of 1, 2-propanediol from a medium copy number vector (pRJ12) was found to be significantly lower than the production by high copy number vector (pRJ11). Hence the medium copy number vector was not selected for 1, 2-propanediol and 1-propanol production.

The result of the *in vivo *enzyme assay (Figure 2) shows almost 100% conversion of 1, 2-propanediol to 1-propanol indicating that the conversion of 1,2-propanediol to 1-propanal was very efficient and that the native expression of alcohol dehydrogenases in *E. coli *is sufficient in converting 1-propanal to 1-propanol. However, it was not the case for the strain carrying the plasmids pRJ11 and pYY93 which showed much lower conversion of 1,2-propanediol to 1-propanol as about 0.46 g/L of 1,2-propanediol was left unconverted. We speculate that the reason for this could be the expression issue of *ppdABC*. The optimal expression of these three subunits can be successfully achieved in aerobic conditions as we did in *in vivo *assay [19]. However, in anaerobic conditions which is required for 1,2-propanediol production, the protein expression might be negatively affected due to low cellular energy and nutrients. Such a problem could be resolved in a more controlled environment such as in a bench scale fermenter by the delicate adjustment of oxygen level during fermentation course.

The accumulation of 7 g/L lactate indicates that the carbon flux towards pyruvate is still strong in anaerobic conditions. The main branch of glycolysis plays the major role. Theoretically, one molecule of fructose-1,6-bisphosphate is broken down into one molecule of glyceraldehyde-3-phosphate and one molecule of dihydroxyacetone phosphate [25]. However, the presence of triose phosphate isomerase seems to channel the carbon flux back to the main branch toward pyruvate biosynthesis [25]. In addition, the pentose phosphate pathway is also very active in low phosphate conditions [26]. This pathway does not generate dihydroxyacetone phosphate as an intermediate, but directly goes to pyruvate. The pyruvate generated is acted upon by lactate dehydrogenase (*ldhA*) resulting in the production of lactate [27]. Another minor route of lactate formation is via the glyoxalase pathway where methylglyoxal is converted to lactate by the native expression of *gloA *[28].

Overall, the work presented here represents 1-propanol production in a wild type *E. coli *strain and forms a basis for further enhancement in production. The effect of competing pathways is significant and the deletion of the same has not been explored in this study. We speculate that by the knock-out of genes encoding for lactate dehydrogenase (*ldhA)*, glyoxalaseI (*gloA*) and other competing pathways (*tpiA *and *zwf*) the production of 1-propanol can be further enhanced, which will be pursued in the near future.

## Materials and methods

### Chemicals and Reagents

Hydroxyacetone was bought from Acros Organics (New Jersey, USA); methylglyoxal and 1,2-propanediol were purchased from Sigma Aldrich (St. Louis, Mo); 1-propanol was obtained from Fisher Scientific (Atlanta, GA). KOD DNA polymerase was obtained from EMD Chemicals Inc., NJ. All restriction enzymes were bought from New England Biolabs (Beverly, MA). The rapid DNA ligase was obtained from Roche Applied Science (Indianapolis, IN). All the enzymes were used according to the instructions of the manufacturer.

### Plasmids and Strains

*E. coli *strain XL1-Blue (Stratagene, CA) was used for DNA manipulations; while wild type *E. coli *strain BW25113 (*E. coli *Genetic Resource Center, CT) and *E. coli *strain BL21* (Invitrogen) were employed for enzyme assays and shake flask experiments. Plasmids pZE12-luc [29], pCS27 [7] and pCDF-Duet1 (EMD Chemicals Inc., NJ) were used for DNA cloning. The features and descriptions of the used strains and plasmids are listed in Table 4.

**Table 4:** List of strains and plasmids used in this study.

Strain	Genotype	Reference
*E. coli *BW25113	*rrnBT14 DlacZWJ16 hsdR514 DaraBADAH33 DrhaBADLD78*	[32]
*E. coli *BL21*	*F^- ^ompT hsdS_B _(r_B_^- ^m_B_^-^) gal dcm (DE3)*	Invitrogen
*E. coli *XL-1 Blue	*recA1 endA1gyrA96thi-1hsdR17supE44relA1lac [F' proAB lacIqZDM15Tn10 (TetR)]*	Stratagene

**Plasmid**	**Description**	**Reference**

pZE12-luc	pLlacO1::luc(VF); *ColE1 ori*; *Amp^R^*	[29]
pCS27	pLlacO1:: MCS; *p15A ori; Kan^R^*	[7]
pCDF-Duet1	pT7lac::MCS;*CDF ori; Sm^R ^*	EMD Chemicals Inc., NJ
pYY93	*ppdABC *from *K. oxytoca *cloned into pCS27	This study
pYY109	*budC *from *K. pneumoniae *cloned into pCDF-Duet1	This study
pYY134	*gldA*BC from *K. pneumoniae *cloned into pCS27	This study
pYY167	*dhab12 *from *C. butyricum *cloned into pCS27	This study
pRJ1	*mgsA *from *C. acetobutylicum *cloned into pZE12-luc	This study
pRJ2	*mgsA *from *B. subtilis *cloned into pZE12-luc	This study
pRJ3	*mgsA *from *C. difficile *cloned into pZE12-luc	This study
pRJ4	*mgsA *from *E. coli *cloned into pZE12-luc	This study
pRJ5	*mgsA *from *T. thermophilus *cloned into pZE12-luc	This study
pRJ6	*mgsA *from *K. pneumoniae *cloned into pZE12-luc	This study
pRJ7	*mgsA *from *P. fluorescens *cloned into pZE12-luc	This study
pRJ8	*mgsA *from *R. eutropha *cloned into pZE12-luc	This study
pRJ9	*gldA *from *E. coli *cloned into pZE12-luc	This study
pRJ10	*ydjG *from *E. coli *cloned into pZE12-luc	This study
pRJ11	*ydjG *from *E. coli, budC *from *K. pneumoniae*, and *mgsA* from *B. subtilis *cloned in pZE12-luc	This study

### DNA manipulations

All DNA manipulations were performed according to the standard procedures as described previously [30]. The primers involved in DNA manipulations are listed in Table 5. The plasmids listed in Table 4 were constructed as described below.

**Table 5:** Primers used in this study.

Plasmid	Gene	Primer Sequence (5'-3')
pRJ1	*mgsA*	F: GGGAAAGGTACCATGGCACTTATAATGAATAGTAAAAAAAAGATAGC R: GGGAAAGCATGCTTAAAAATTGTCTTTTCTAATTTTTTGGTAATAAT
pRJ2	*mgsA*	F: GGGAAAGGTACCATGAAAATTGCTTTGATCGCGCATG R: GGGAAAGCATGCTTATACATTCGGCTCTTCTCCCCGA
pRJ3	*mgsA*	F: GGGAAAGGTACCATGAATATAGCATTAGTAGCACATGACCAAATGAA R: GGGAAAGCATGCTTAAATACGTTGACTTTTGCTTTTTCTAACTTCTC
pRJ4	*mgsA*	F: GGGAAAGGTACCATGGAACTGACGACTCGCAC R: GGGAAAGCATGCTTACTTCAGACGGTCCGCGA
pRJ5	*mgsA*	F: GGGAAAGGTACCATGCCCATGAAGGCCCTGGC R: GGGAAAGCATGCCTATTGGGGGGTTCCCTTGC
pRJ6	*mgsA*	F: GGGAAAGGTACCATGTGGAATGAAAATATGGAACTGACAACACGTAC R: GGGAAAGCATGCTTATTTCAGGCGCTCGGCAA
pRJ7	*mgsA*	F: GGGAAATGTACAATGATCGGTATCAGTTTCACCC R: GGGAAAGCATGCTTATCCTCGGCCGGCCAGGTA
pRJ8	*mgsA*	F: GGGAAAGGTACCATGACTCGCCCCCGCATCGCGTTGAT R: GGGAAATCTAGATCAGCTGGCCGCCGCTTCGT
pRJ9	*gldA*	F: GGGAAAGCATGC*AGGAGATATACC*ATGGACCGCATTATTCAATCACCGG R: GGGAAATCTAGATTATTCCCACTCTTGCAGGAAACGC
pRJ10	*ydjG*	F: GGGAAAGGTACCATGAAAAAGATACCTTTAGGCACAACGG R: GGGAAATCTAGATTAACGCTCCAGGGCCTCTGCCATTTCC
pRJ11	*ydjG* *budC* *mgsA*	F: GGGAAAGGTACCATGAAAAAGATACCTTTAGGCACAACGG R: GGGAAAGTCGACTTAACGCTCCAGGGCCTCTGCCATT F: GGGAAAGTCGAC*AGGAGATATACC*ATGAAAAAAGTCGCACTTGTTACCGG R: GGGAAACTGCAGTTAGTTAAACACCATCCCGCCGTCG F: GGGAAACTGCAG*AGGAGATATACC*ATGAAAATTGCTTTGATCGCGCATGAC R: GGGAAATCTAGATTATACATTCGGCTCTTCTCCCCGA
pYY93	*ppdABC*	F: GGGAAACGTACGATGAGATCGAAAAGATTTGAAGCACTGGCGAAACG R: GGGAAAAAGCTTTTAATCGTCGCCTTTGAGTTTTTTACGCTCGACG
pYY109	*budC*	F: GGGAAAGGATCCGAAAAAAGTCGCACTTGTTACCGGCG R: GGGAAAGTCGACTTAGTTAAACACCATCCCGCCGTCG
pYY134	*gldABC*	F: GGGCCCGGTACCATGAAAAGATCAAAACGATTTGCAGTACTGGCCCA R: GGGCCCAAGCTTTTAGCTTCCTTTACGCAGCTTATGCCGCTGCTGAT
pYY167	*dhaB12*	F: GGGAAAGGTACCATGATCAGCAAAGGGTTCAGCACCCAG R: GGGAAAAAGCTTTTATTCCGCGCCTATAGTACACGGAATGCCCATAA

Underlined nucleotides represent restriction sites. Italicized nucleotides represent ribosome binding sites inserted in the primer.

For the methylglyoxal synthase assay, the plasmids pRJ1-pRJ8 were constructed by cloning *mgsA *genes from eight different sources into the vector pZE12-luc separately. Using the primers listed in Table 5, the *mgsA *genes were PCR amplified from the genomic DNA of *C. acetobutylicum *(ATCC824), *B. subtilis *168, *Clostridium difficile *R20291, *E. coli *MG1655, *Thermus thermophilus *HB27, *K. pneumoniae *MGH78578, *Pseudomonas fluorescens *Pf-5, and *Ralstonia eutropha *H16 respectively. The DNA fragments obtained were digested with restriction enzymes for three hours. *Acc65I *and *SphI *restriction enzymes were used to digest *mgsA *genes from *C. acetobutylicum *(ATCC824), *B. subtilis *168, *C. difficile *R20291, *E. coli *MG1655, *T. thermophilus *HB27, *K. pneumoniae *MGH78578, *BsrGI *and *SphI *for the *mgsA *from *P. fluorescens *Pf-5, and *Acc65I *and *XbaI *for the *mgsA *from *R. eutropha *H16. The vector pZE12-luc was also digested with the appropriate restriction enzymes for the above mentioned eight genes. The digested genes were then inserted into the vector pZE12-luc separately.

In order to determine the activity of methylglyoxal reductase, the plasmid pRJ10 was constructed. The *ydjG *gene PCR amplified from *E. coli *MG1655 was cloned into pZE-12luc with restriction enzymes *Acc65I *and *XbaI *generating pRJ10. For the assay of secondary alcohol dehydrogenases, plasmids pRJ9 and pYY109 were constructed. The *gldA *gene from *E. coli *MG1655 was inserted into pZE12-luc vector using restriction enzymes *SphI *and *XbaI *for the construction of plasmid pRJ9. We created pYY109 by inserting the *budC *gene from *K. pneumoniae *MGH78578 into pCDF-Duet1 vector. The restriction enzymes used for the construction of plasmid pYY109 were *BamHI *and *SalI*.

For diol dehydratase assay, plasmids pYY93, pYY134, and pYY167 were constructed. The *ppdABC *operon obtained via PCR from the genomic DNA of *K. oxytoca *was digested with restriction enzymes *BsiWI *and *HindIII *and inserted into plasmid pCS27 digested by *BsiWI *and *HindIII*. Similarly, the *gldABC *operon was PCR amplified from genomic DNA of *K. pnuemoniae *MGH 78578 and inserted into pCS27 using restriction enzymes *Acc65I *and *HindIII*. To construct pYY167, we first synthesized the operon *dhaB12 *from *Clostridium butyricum *by a PCR assembly of 50 bp oligonucleotides designed from Helix Systems (NIH). The codons were optimized for *E. coli *standard expression. The operon was cloned into pCS27 using *Acc65I *and *HindIII*, forming pYY167.

Following the enzyme assays, plasmid pRJ11 was constructed using the most active enzymes in order to produce 1, 2-propanediol. The plasmid pRJ11 was generated via the ligation of three genes on the backbone of pZE12-luc vector. The genes *ydjG*, *budC *and *mgsA *were PCR amplified using the primers listed in Table 5 from the genomic DNA of *E. coli *MG1655, *K. pnuemoniae *MGH78578 and *B. subtilis *168, respectively. Following this, the PCR amplified *ydjG *gene product was digested with *Acc65I *and *SalI*. The PCR amplified *budC *gene product was digested with *SalI *and *PstI*, and the PCR amplified *mgsA *gene was digested with *PstI *and *XbaI*. Vector pZE12-luc was digested with *Acc65I *and *XbaI*. After digestion for three hours, the three gene fragments and the vector were ligated simultaneously, creating pRJ11. It should be noted that RBS sequence (AGGAGA) was inserted upstream of each structure gene with 6-8 nucleotides in between to facilitate protein translations.

### Culture Medium and Fermentation Conditions

M9 minimum was used for the in-vivo assay of propanediol dehydratase and low-phosphate minimum medium was used for shake flask fermentations. M9 minimum media consisted of (per liter): 20 g glucose, 5 g yeast extract, 12.8 g Na_2_HPO_4_.7H_2_O, 3 g KH_2_PO_4_, 0.5 g NaCl, 1 g NH_4_Cl, 0.5 mM MgSO_4_, and 0.05 mM CaCl_2. _The low-phosphate media consisted of (per liter): 20 g glucose, 5 g NaCl, 5 g yeast extract, 1.5 g KCl, 1 g NH_4_Cl, 0.2 g MgCl_2_, 0.07 g Na_2_SO_4_, and 0.005 g FeCl_3_, which was buffered to pH 6.8 with 13.3 g of NaHCO_3 _and 10 g of 3-[N-morpholino] propanesulfonic acid (MOPS) [10,12].

For the shake flask fermentations, 1 mL of seed culture was prepared in LB media containing necessary antibiotics and grown overnight at 37°C in a shaker set at 250 rpm. After overnight incubation, the culture was inoculated into 20 mL of M9 or low-phosphate media containing appropriate antibiotics in 150 mL serum bottles. After growing at 37°C for 3 hours, the cultures were switched to an anaerobic condition by sparging nitrogen gas. IPTG was added into the culture to a final concentration of 0.1 mM 6 hours after inoculation to induce protein expression. Then the fermentation was carried out at 30°C at 250 rpm. Samples were taken after 24 and 48 hours and analyzed with HPLC-RID.

### HPLC-RID Analysis

The analysis of fermentation products was done via HPLC (Shimadzu) equipped with a Coregel-64H column (Transgenomic). 1 mL sample was collected and centrifuged at 15,000 rpm for 10 minutes and the supernatant was filtered and used for analysis. The mobile phase used was 4 mN H_2_SO_4 _having a flow rate of 0.6 mL/min and an oven temperature set at 60°C [31].

### Enzyme Crude Extract Preparation

*E. coli *strain BL21* was employed to express *budC *carried by pYY109. Expression of other individual enzymes was conducted in the wild type *E. coli *strain BW25113 harboring the corresponding plasmids. Generally, the transformed strains were pre-inoculated into LB liquid medium containing appropriate antibiotics and grown at 37°C overnight with shaking. The following day, 1 mL of preinoculum was added to 50 mL of fresh LB medium containing necessary antibiotics. The culture was left to grow at 37°C with shaking until the OD_600 _reached approximately 0.6. At that point, IPTG was added to a final concentration of 1 mM and the protein expression was conducted at 30°C for 3 hours. The cells were collected by centrifugation at 5000 rpm for 10 min at 4°C. The cell pellets were resuspended in 2 mL of 50 mM imidazole-HCl buffer (pH 7.0). Cell disruption was performed using French Press, and soluble protein was obtained by ultra-centrifugation for enzyme assays. Total protein concentration was estimated using the BCA kit (Pierce Chemicals).

### Methylglyoxal Synthase Assay

Methylglyoxal synthase assay was performed as described previously with minor revisions [10,11,23]. The assay was carried out using a two step procedure. The reaction mixture (500 μl) consisted of 50 mM imidazole-HCl buffer (pH 7.0), 0.15-1.5 mM dihydroxyacetone phosphate, and 25 μl crude extract. Reaction was started with the addition of 25 μl crude extract to the reaction mixture and incubated in a water bath at 30°C for 30 seconds. Followed by this, the reaction was immediately stopped by the addition of 30 μl sample of the reaction mixture to the detection mixture and incubated in a water bath at 30°C for 15 minutes. The detection mixture consisted of 300 μl of DI water, 110 μl of 0.1% 2, 4-dinitrophenylhydrazine dissolved in 2N HCl. After the completion of 15 minutes 550 μl of 10% NaOH was added to the detection mixture and then incubated at room temperature for another 15 minutes. (Final volume of detection mixture = 990 μl). The samples were diluted 10 times before measuring the absorbance at 550 nm. 1 μmol of methylglyoxal has an absorbance value of 16.4 at 550 nm [11].

### Methylglyoxal reductase assay

The reaction mixture contained 20-120 mM methylglyoxal and 0.25 mM NADH, in imidazole-HCl buffer (pH 7.0) having a final volume of 970 μl. The assay was begun with the addition of 30 μl crude extract to the reaction mixture. The reaction was allowed to proceed for 60 seconds at 37°C. We measured the decrease in absorbance of NADH at 340 nm to calculate the specific activity [10,11].

### Secondary Alcohol Dehydrogenase Assay

The enzyme crude extracts prepared from *gldA *and *budC *expression were used for this assay. The reaction mixture consisted of 20-120 mM of methylglyoxal or hydroxyacetone and 0.25 mM NADH in imidazole-HCl buffer at (pH 7.0) with a final volume of 970 μl. The assay was begun with the addition of 30 μl crude extract to the reaction mixture. The reaction was allowed to proceed for 60 seconds at 37°C. We measured the decrease in absorbance of NADH at 340 nm to calculate the specific activity [10,11].

### Propanediol Dehydratase *in vivo *Assay

The assay was carried out to evaluate the activities of three diol dehydratases on 1,2-propanediol. Three *E. coli *strains generated by transforming the wild type *E. coli *BW25113 with pYY93, pYY134, and pYY167 respectively were used for this purpose. Preinoculum from an overnight culture was added to 10 mL of M9 media (1:100 V/V) and grown at 37°C. IPTG was added to the cultures to a final concentration of 0.1 mM and 1,2-propanediol was added to the cultures as the substrate to a final concentration of 5 g/L (65.7 mM) after 4 hours. The cell cultures carrying pYY167 was grown anaerobically; while the cell cultures carrying pYY93 or pYY134 were grown micro-aerobically. Coenzyme-B12 (cobamamide) was also added to the cell cultures having pYY93 and pYY134 to a final concentration of 10 μM after 4 hours. Samples were collected after 24 hours and analyzed for 1-propanol generation using HPLC-RID as described above. The enzyme activities were reflected by the formation of 1-propanol.

## Competing interests

The authors declare that they have no competing interests.

## Authors' contributions

YY and RJ conceived the study. RJ performed the experiments under the guidance of YY. An equal contribution by YY and RJ was made for literature review and drafting of the manuscript. Both authors read and approved the final manuscript.
