# Correction: dehydratase mediated 1-propanol production in metabolically engineered *Escherichia coli*

**DOI:** 10.1186/1475-2859-11-38

**Published:** 2012-03-30

**Authors:** Rachit Jain, Yajun Yan

**Affiliations:** 1 Biochemical Engineering Program, Faculty of Engineering, 601B Driftmier Engineering Center, University of Georgia, Athens, GA 30602, USA

## Correction

After publication of this work [1], we have noticed accidental errors that were introduced during our revision process. In our revision process, to address review comments we reduced the number of significant digits for the results of our enzyme assay experiments. As we reduced the number of significant digits to two in Tables 1 and 2, we overlooked the corresponding values mentioned in the text of "Results and Discussion" section discussing the "Methylglyoxal Synthase Assay" and "Secondary Alcohol Dehydrogenase Assay" [1]. In order to maintain consistency between the text and the tables, we would like to correct Tables 1 and 2 by increasing the number of significant digits up to four, which were used for our original submission. These changes will in no manner affect the outcome/interpretation of the experiments as described in the original publication and will not affect the merit of this work. In addition, we would like to modify the "Competing Interests" as below. The authors apologize for any inconvenience caused thereof.

**Table 1:** Methylglyoxal synthase assay results

*mgsA *source	Specific Activity (U/mg)	*K_m _*(mM)	Specific Activity/*K_m _*(U/mg/mM)
*C. acetobutylicum*	0.0541 ± 0.0042	0.776 ± 0.005	0.0697

***B. subtilis***	**0.0561 **± **0.0031**	**0.473 **± **0.070**	**0.1186**

*C. difficile*	0.0597 ± 0.0039	1.439 ± 0.060	0.0415

*E. coli*	0.1242 ± 0.0069	1.418 ± 0.120	0.0876

*T. thermophilus*	0.0161 ± 0.0004	2.118 ± 0.070	0.0076

*K. pneumoniae*	0.0165 ± 0.0009	2.820 ± 0.300	0.0058

*P. fluorescens*	0.0133 ± 0.0082	1.560 ± 0.020	0.0085

*R. eutropha*	0.0052 ± 0.0004	0.700 ± 0.030	0.0074

Substrate dihydroxyacetone phosphate concentration was varied from 0.15 mM to 1.5 mM for all reactions. 1 unit (U) was defined as the amount (μmoles) of methylglyoxal formed per unit time (min).

**Table 2:** Specific activity and *K_m _*determination of the secondary alcohol dehydrogenases

Gene	Methylglyoxal		Hydroxyacetone	
	
	Specific Activity (U/mg)	*K_m_* (mM)	Specific Activity (U/mg)	*K_m_* (mM)
*gldA*	2.456 ± 0.001	68.24 ± 0.05	0.912 ± 0.008	10.47 ± 0.55

*budC*	3.718 ± 0.066	0.78 ± 0.03	4.970 ± 0.007	1.83 ± 0.63

The decrease in absorbance of NADH at 340 nm was recorded and used for calculations using the substrates methylglyoxal and hydroxyacetone. Substrate concentration was varied from 20 mM - 120 mM. 1 unit (U) was defined as the amount (μmoles) of product formed per unit time (min).

## Competing interests

The University of Georgia has filed a United States provisional patent on this technology.

## Authors' contributions

YY and RJ conceived the study. RJ performed the experiments under the guidance of YY. An equal contribution by YY and RJ was made for literature review and drafting of the manuscript. Both authors read and approved the final manuscript.
